# Concentration of Antifouling Biocides and Metals in Sediment Core Samples in the Northern Part of Hiroshima Bay

**DOI:** 10.3390/ijms15069991

**Published:** 2014-06-04

**Authors:** Noritaka Tsunemasa, Hideo Yamazaki

**Affiliations:** 1 Environmental Conservation Division of the Environment Bureau, Hiroshima City, 1-6-34 Kokutaiji-Machi, Naka-ku, Hiroshima 730-8586, Japan; 2 School of Science and Engineering, Department of Life Science, Kinki University, 3-4-1 Kowakae, Higashiosaka, Osaka 577-8502, Japan; E-Mail: yamazaki@life.kindai.ac.jp

**Keywords:** Irgarol 1051, M1, Diuron, Sea-Nine 211, metal, sediment core, chronology

## Abstract

Accumulation of Ot alternative antifoulants in sediment is the focus of this research. Much research had been done on surface sediment, but in this report, the accumulation in the sediment core was studied. The Ot alternative antifoulants, Diuron, Sea-Nine211, and Irgarol 1051, and the latter’s degradation product, M1, were investigated in five samples from the northern part of Hiroshima Bay. Ot compounds (tributyltin (TBT) and triphenyltin (TPT)) were also investigated for comparison. In addition, metal (Pb, Cu, Zn, Fe and Mn) levels and chronology were measured to better understand what happens after accumulation on the sea floor. It was discovered that Ot alternative antifoulant accumulation characteristics in sediment were like Ot compounds, with the concentration in the sediment core being much higher than surface sediment. The concentration in sediment seems to have been affected by the regulation of Ot compounds in 1990, due to the concentration of Ot alternative antifoulants and Ot compounds at the survey point in front of the dock, showing an increase from almost the same layer after the regulation.

## 1. Introduction

Organotin (Ot) compounds, used for many years as antifouling biocides on ships, marine structures and fishing nets, became a problem, because of their toxicity and environmental accumulation [1].

The movement towards global regulation began in October 2001, when the International Maritime Organization (IMO) adopted the International Convention on the Control of Harmful Antifouling Systems (AFS Convention) [2]. The regulation took effect on the 17 September 2008, prohibiting the use of Ots as active ingredients in antifouling agents on marine vessels. Therefore, instead of the prohibited Ot compounds, paint manufacturers had to develop many alternative products to be used as antifouling coatings.

At first, it was thought that Ot alternative antifoulants would not accumulate as much in sea water and sediment as organotin compounds, because the resolution speed of Ot alternative antifoulants was faster [3]. However, Ot alternative antifoulants have subsequently been detected at higher levels than initially expected in sea water and sediment, which has caused concern about the possible effects.

The presence of Ot alternative antifoulants in surface sediment had previously been researched in areas, such as Hong Kong [4], India [5], Malaysia [6], Thailand [7], Vietnam [8], Greece [9], Denmark [10], *etc.* However, little was known about the accumulation of these compounds in the sediment core. The detection of biocides in the sediment core in earlier studies in the United Kingdom [11], Brazil [12], Korea [13], Japan [14], *etc.*, were only of organotin compounds. In Korea, Brazil and Japan, the highest concentration of tributyltin (TBT) was detected at 46,100, 6000 and 962 µg/kg, respectively.

In this report, the Ot alternative antifoulants, Diuron, Sea-nine211 and Irgarol 1051, and the latter’s degradation product, M1, were investigated in five sediment core samples taken from the northern part of Hiroshima Bay. Ot compounds were also investigated for comparison with alternative antifoulants. In addition, metal (Pb, Cu, Zn, Fe and Mn) levels and chronology were measured to better understand what occurs after metals accumulate in the sediment core.

## 2. Results

### 2.1. Alternative Biocides

**Figure 1:**
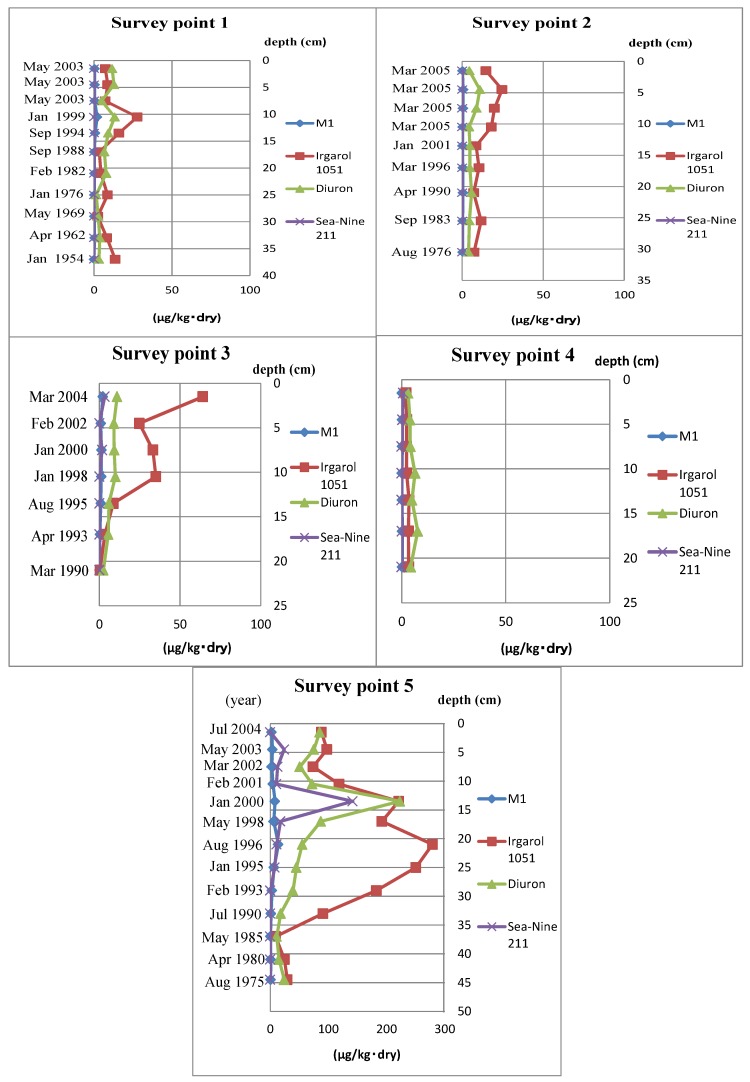
Concentrations of organotin alternative antifoulants in sediment core samples. The survey Point 4 sediment year was not measuredin sediment core samples from Hiroshima Bay, (Hiroshima, Japan). Sediment years were calculated by the ^21^^0^Pb concentration.

In this study, all of the investigated compounds were detected in sediment core samples. The detected ranges of the investigated compounds are shown in Table 1. Every depth’s detected concentration in the sediment core samples is shown in Figure 1. Accumulation values of all investigated compounds except M1 were obtained.

Previous data on Ot alternative antifoulants in sediment core samples could not be found, so the results of this research were compared with that of previous reports on sea floor sediment samples instead. The antifouling biocide and degradation product concentration levels in previous samples are shown in Table 2.

The residual of Irgarol 1051 in sediment samples was reported by many researchers. The highest levels of Irgarol 1051 detected in sea floor sediment samples from harbors in the UK, Greece, the Baltic Sea, Japan’s Otsuchi Bay, Spain, the North Sea and Japan’s Osaka Bay were 1011 µg/kg [15,16,17,18,19], 690 µg/kg [20], 220 µg/kg [21], 100 µg/kg [22], 57 µg/kg [23], 14 µg/kg [21] and 8.2 µg/kg [24], respectively. In the survey Point 5 sediment core sample, the highest concentration level was measured at 270 µg/kg, making it the third highest level behind the U.K. and Greek sea floor sediment samples.

**Table 1:** Minimum-maximum concentrations of investigated compounds in sediment core samples from Hiroshima Bay, (Hiroshima, Japan).

Point Number	Irgarol 1051	M1	Diuron	Sea-Nine 211
1	2.4–27	ND–0.8	1.4–13	ND
2	7.1–24	0.2–0.5	3.9–10	ND
3	0.4–64	0.1–2.1	2.5–11	ND–3.3
4	2.1–3.5	0.1–0.3	3.0–7.7	ND–0.8
5	9.1–270	0.4–13	11–220	7.6–140

ND: not detected; Unit: μg/kg∙dry.

**Table 2:** Previous Irgarol 1051, M1, Diuron and Sea-Nine 211 research data.

Location	Concentrations (µg/kg)				Reference
	Irgarol 1051	M1	Diuron	Sea-Nine 211	
Hiroshima Bay, Japan (2004, 2005)	0.4–270	<0.1–13	1.4–220	<1.0–140	this research
Hiroshima Bay, Japan (2002–2005)	<1–28	<1–9	<4–73	***	[25]
Otsuchi Bay, Japan (2005)	<0.05–100	<0.18–0.47	<0.08–530	<0.04–150	[22]
Osaka Bay, Japan (2003)	<0.08–8.2	<0.18–2.9	<0.64–1350	<0.04–2.4	[24]
Piraeus-Elefsina, Greece (1999, 2000)	<LOD–690	***	***	<LOD–49	[20]
Southampton Water, UK (2000)	0.3–3.5	<LOD–0.3	0.4–6.2	***	[15]
Barcelona, Spain	3–57	0.2–3.3	***	***	[23]
Southampton Water, UK (1998)	<LOD–110	***	<LOD–1420	***	[16]
Baltic Sea (1997, 1998)	<4–220	***	***	***	[21]
North Sea (1997, 1998)	<LOD–14	***	***	***	[21]
Orwell Estuary, UK (1998)	<10–1011	***	<12–395	***	[17]
The Solent, UK (1998)	<LOD	<LOD–5.7	<LOD	***	[26]
Blackwater Estuary, UK (1998, 1999)	3.3–222	***	***	***	[18]
Hamble Estuary, UK	12–190	***	***	***	[19]

LOD: limit of detection; ***: not measured.

The highest concentration of the Irgarol 1051 degradation product, M1, in sediment was detected at 5.7 µg/kg in the UK harbors, the Solent, (Isle of Wight, UK) [15,26], 2.9 µg/kg in Osaka Bay [24] and 0.47 µg/kg in Otsuchi Bay (Iwate Pref., Japan) [22]. In comparison, the highest level of concentration in survey Point 5 sediment was significantly higher at 13 µg/kg.

The highest concentration of Diuron in sediment detected in the U.K. was 1420 µg/kg in Southampton water (Hampshire, UK) [15,16,17]. In Japan, Osaka Bay measured at 1350 µg/kg [24] and 530 µg/kg in Otsuchi Bay [22]. The Hiroshima Bay results indicated that the highest concentration level was in survey Point 5 sediment at 220 µg/kg, making it lower than the sea floor sediment samples of other reported data.

Surprisingly, the concentration of Sea-Nine 211 in sediment had only been reported in a few publications. From the few articles that do list it, the highest concentration of Sea-Nine 211 in sediment was reported at 150 µg/kg in Otsuchi Bay [22], 49 µg/kg in Piraeus-Elefsina, (Athens, Greece) [20], and 2.4 µg/kg in Osaka Bay [24]. The highest concentration level in survey Point 5 sediment was 140 µg/kg, approximately the same as in Otsuchi Bay.

### 2.2. Organotin Compounds

**Figure 2:**
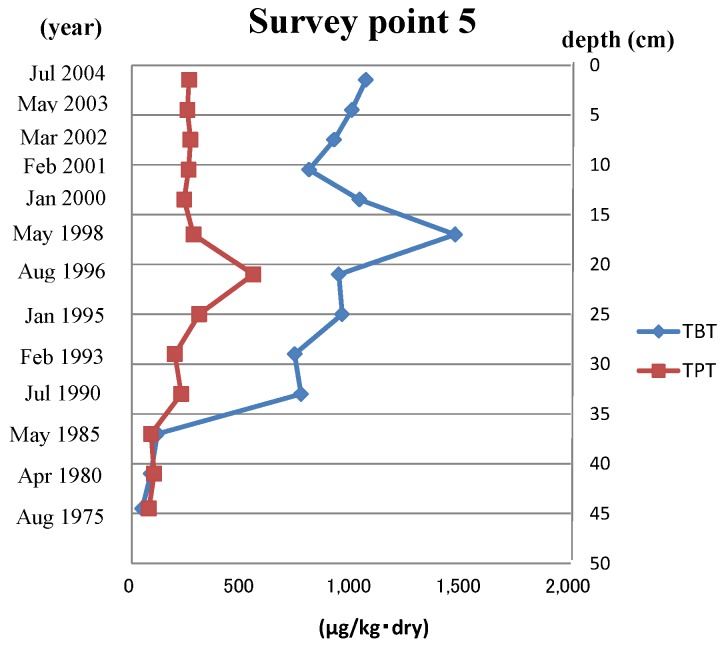
Concentrations of organotin compounds in survey Point 5 sediment core samples. The sediment years were calculated by the ^21^^0^Pb concentration. TBT, tributyltin; TPT, triphenyltin.

Ot compounds (TBT and triphenyltin (TPT)) were only investigated at survey Point 5, where the Ot compound’s value had been high in previous research. TBT and TPT were detected from 48 to 1400 µg/kg and from 75 to 550 µg/kg, respectively. The analytical results of survey Point 5 are also shown in Figure 2.

In Korea, the highest concentration of TBT in the sediment core sample, which was gathered in Ulsan Bay nearby a maintenance shipyard and in Okpa Bay nearby a shipyard, were 46,100 µg/kg and 9000 µg/kg, respectively [13]. In Brazil, the highest concentration of TBT in the sediment core sample gathered from Guanabara Bay, home of the second most important trading harbor in Brazil, was 6000 µg/kg [12]. The highest concentration level of TBT, which was detected at 962 µg/kg [14] in survey Point 5 was considerably lower than in the Korean and Brazilian samples. The large numbers of vessels and the lack of regulation on the use of TBT in Korea and Brazil could explain the much higher levels of TBT recorded in these areas.

### 2.3. Metals

**Figure 3:**
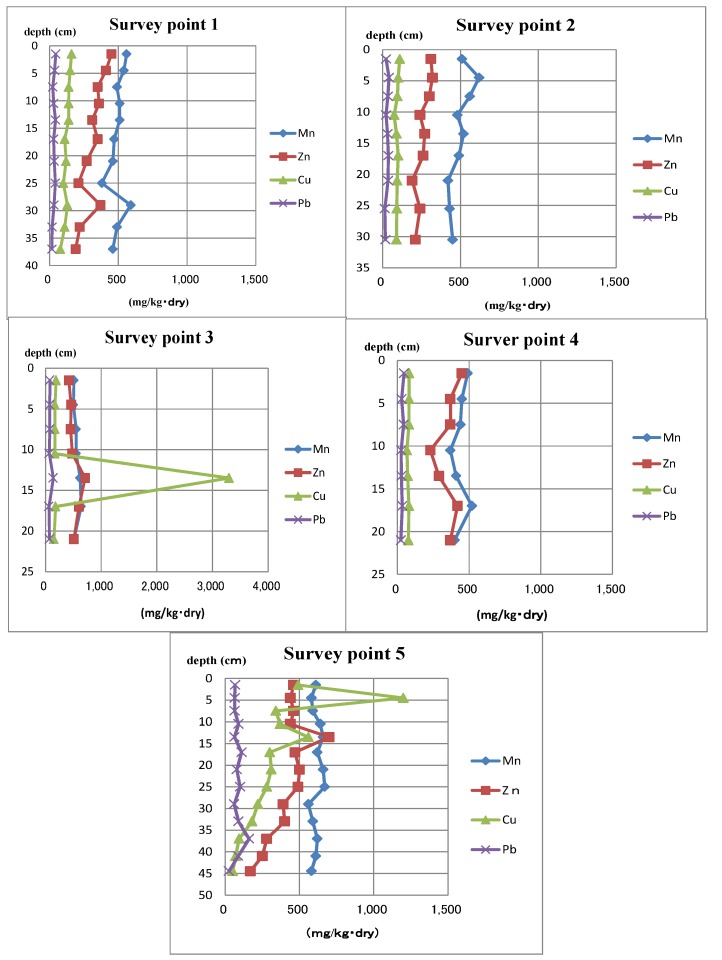
Concentrations of metals in sediment core samples.

The detected ranges of metals are shown in Table 3. Every depth’s detected concentrations of sediment core samples are shown in Figure 3. This analysis was performed to gather data on the concentrations of metals along with how they reacted to oxidation and the reduction of sediment. Generally, accumulation is shown by measuring Mn, Fe and Cu. 

**Table 3:** Minimum-maximum concentrations of metals.

Point Number	Pb	Cu	Zn	Fe	Mn
1	17–45	78–160	190–450	36,000–50,000	380–590
2	14–41	74–110	190–320	37,000–46,000	430–620
3	62–140	150–3300	420–710	60,000–82,000	500–640
4	25–47	67–82	230–450	48,000–60,000	370–520
5	25–160	50–1200	170–700	41,000–45,000	560–670

Unit: mg/kg∙dry.

In this research, it was only Cu that showed signs of accumulation. At Point 3, in the 12–15 cm layer, Cu was detected at 3300 mg/kg. At Point 5 in the 3–6 cm layer, 1200 mg/kg was detected. Furthermore, at Point 5, Cu was detected in the 12–15 cm layer at 600 mg/kg and Zn was detected in the same layer at 750 mg/kg.

## 3. Discussion

In general, chemical compounds are often attached to the smaller particles. The grain distribution of the sediment core’s surface is shown in Table 4.

Survey Points 1 and 2 were located in the same fishery harbor. Point 2 is nearer than Point 1 with respect to its entrance, so the specific surface area, below 67 µm (%), of Point 2 is lower than Point 1. However, their concentration level was almost the same. It was thought that many leisure boats anchored in the fishery harbor long term.

Though the specific surface area, below 67 µm (%), of Point 4 was relatively high, the concentration of Ot alternative antifoulants was much lower, due to the boats only passing through the survey point.

Survey Point 5 was mostly high in specific surface area and below 67 µm (%) in all samples. The Ot alternative antifoulants were thought to have come from the nearby dock, so it is believed that the accumulation speed was high.

**Table 4:** Grain distribution of the sediment core’s surface.

Survey Point	Specific Surface Area (cm^2^/cm^3^)	Median Particle Size (µm)	Arithmetic Mean Particle Size (µm)	Below 67 µm (%)
1	33,926	10.1930	15.0119	97.5
2	24,890	14.4307	33.6879	86.0
3	33,374	10.1875	19.3025	93.4
4	37,229	8.9690	15.4780	95.7
5	40,214	7.6898	12.1456	98.1

Grain distribution was mesured by HORIBA LA-920 (HORIBA K.K., Kyoto, Japan).

The concentration of antifouling biocides in sediment core samples from Hiroshima Bay was much higher than the corresponding sea floor sample concentrations [25]. In particular, the survey Point 5 sample had the highest observed concentration of the alternative biocides.

As shown in Figure 1 and Figure 2, the concentration of Ot alternative antifoulants and Ot compounds at survey Point 5 began to increase from almost the same point. In the case of Ot alternative antifoulants, it was thought that the rise in the concentration was caused by the increase of the consumption after the regulation of Ot compounds in 1990. On the other hand, in the case of Ot compounds, the cause was mainly due to the way the dock replaced the Ot compound paints with the Ot alternative antifoulants. When the regulation became effective in Japan, the Ot compounds were stripped from ships before the Ot alternative antifoulants were painted on to the ships. In the case of this dock, the stripped pieces of Ot compound paint were not cleared from the dry dock, so when the ships were relaunched, the stripped paint chips on the ground were washed into the sea water. Obviously, this would have had an impact on the Ot compound concentration levels shown in Figure 2.

The sediment core’s layers were thought to indicate the chronology of the biocide’s accumulation. At first, it was thought that the concentration of biocides would be higher in the layers closest to the surface, with a gradual decrease in the deeper layers. In fact, the results indicated differently, as the highest concentration levels were detected at deeper points. Metals in the sediment, especially Mn, Fe and Cu, are known to go through a process of dissolution as the sediment conditions cycle through oxidation and reduction, as new layers of sediment form [27].

Metal concentrations in every core section were measured to elucidate the distribution in every layer and their relationship to the concentrations of biocides analyzed. In this study, only the concentrations of Cu, which were obtained at survey Points 3 and 5 showed signs of accumulation. However, it was thought that this accumulation was not as a result of oxidation and reduction, due to accumulation of Mn or Fe not being detected. Both Mn and Fe generally react more to oxidation and reduction than Cu, making it unlikely that oxidation and reduction were the cause of accumulation. Furthermore, the increase of Zn in the same layer with a similar pattern to Cu support this theory. The more likely cause of Cu accumulation is that Cu was present in the paint fragments of the antifouling biocides. Therefore, from this research, it is inconclusive whether or not alternative antifouling biocides are affected by the change in conditions due to oxidation and reduction.

According to Jacobson *et al.* [3], the half-life of Sea-Nine 211 and TBT in sea water is one hour and six to nine months, respectively. It was thought that Sea-Nine 211 did not accumulate in sediment. However, high concentrations were measured in sediment cores. Therefore, it is clear that the binding to sediment protects molecules from breakdown.

Furthermore, according to Tsunemasa *et al.* [28,29], the toxicity (LC_50_) of Sea-Nine 211 and TBT on oyster embryos is 28 and 16 µg/L, respectively. The highest value of Sea-Nine 211 and TBT in the survey Point 5 sediment core is five and 87 times more compared with the toxicity values on oyster embryos, respectively. If these compounds dissolve in sea water, it is clear that they have an effect on the marine organisms (for example, oyster and sea urchin embryos).

As the consumption of these biocides increases, it will have a negative impact through the retardation of the development of eggs and embryos, leading to an increased mortality rate. Therefore, it is thought that the antifouling biocide resolution speed in sea water and sediment should be improved.

## 4. Experimental Section

### 4.1. Sampling Description

**Figure 4:**
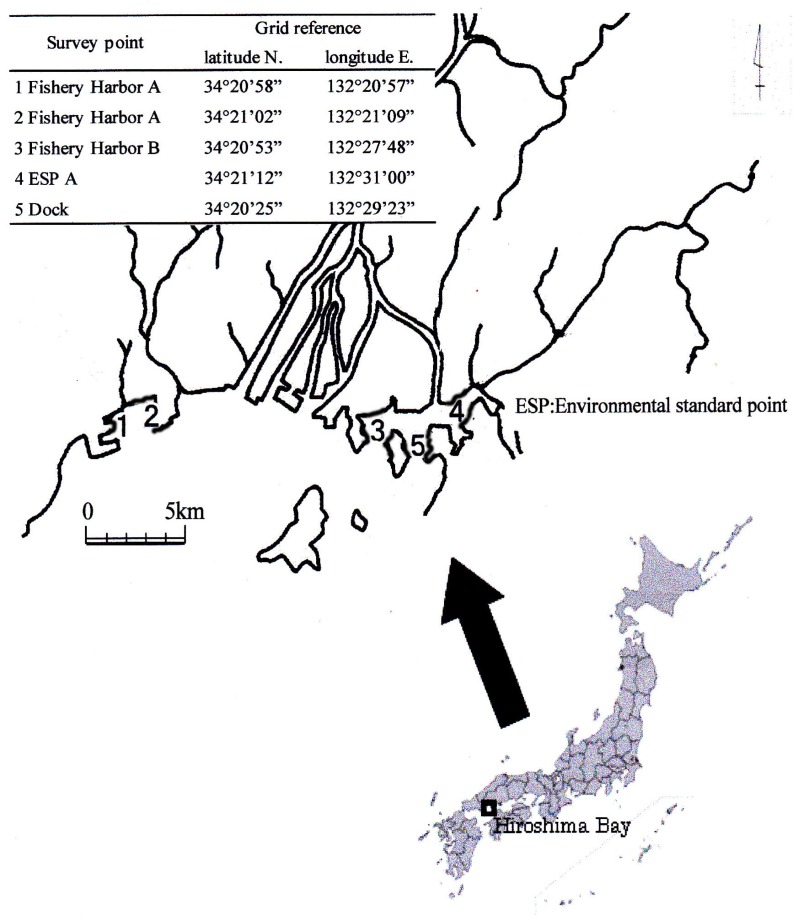
Survey points.

A map of the sampling stations is shown in Figure 4. Three fishery harbors, one environmental standard point and one dock around the Otagawa River mouth were investigated. Five sediment samples were collected in December 2004 and March 2005. At Points 1 and 2 in the fishery harbor, there were many more leisure boats than commercial fishing boats anchored. At Point 3 in the fishery harbor, the ratio of commercial fishing boats to leisure boats was about the same. The environmental standard Point 4 was located in a recess of Hiroshima Bay. Point 5 was located at the entrance to the dock, and it was at this point where high concentrations of organotin compounds had been previously reported.

#### 4.1.1. Sampling

The sediment from the sea floor was collected with a sediment core sampler, divided into 3–5 cm-thick sections, centrifuged at 3000 rpm for 10 min and stored at −20 °C until analysis. Survey Points 1 and 2 were collected on 19 December 2004, other points were collected on 1 March 2005. The collected depths were 39, 33, 23, 23 and 46 cm, respectively.

#### 4.1.2. Chemicals

The investigated materials were Diuron (3-(3,4-dichlorophenyl)-1,1-dimethylurea), Sea-Nine211^®^ (4,5-dichloro-2-*n*-octyl-3(2H)isothiazolone) (Rhom and Hass, Philadelphia, PA, USA), Irgarol 1051^®^ (2-methylthio-4-*tert*-butylamino-6-cyclopropylamino-*s*-triazine) (Chiba Speciality Chemicals K.K., Basel, Switzerland) and the latter’s degradation product, M1 (2-methylthio-4-*tert*-butylamino-6-amino-*s*-triazine), which were thought to be commonly used in Japan as biocides. The Ot compounds (TBT and TPT) were also investigated for comparison with the alternative antifoulants. In addition, metals (Pb, Cu, Zn, Fe and Mn) in the core samples were measured in order to detect the behavior that was caused by the change in the condition of oxidation and reduction.

### 4.2. Chemical Analysis

#### 4.2.1. Alternative Biocides

The method used for the determination of alternative compounds in sediment was based on that used by Harino *et al.* [24], with some modification. In a centrifuge tube, 5 g of wet sediment were mixed with 10 mL of acetonitrile for 10 min with a mechanical shaker. After removing the supernatant, the analytes were re-extracted with 10 mL of acetonitrile for 10 min, and then the mixture was centrifuged. The combined supernatants were concentrated to about 1 mL on a rotary evaporator, and then, up to 45 mL of distilled water was added. The analytes were then extracted three times with 10 mL of dichloromethane, and the organic layer was dried with anhydrous Na_2_SO_4_. After 10 mL of methanol and 100 µL of atrazine-_13_C_3_ (1 mg/L) were added to the organic layer, which was then concentrated to 2 mL by rotary evaporation. The analytes were detected by LC/MS-MS. The analytical condition is shown in Table 5. The detection limits of Sea-Nine 211, Diuron, Irgarol 1051 and M1 in the sediment samples were set at 1.0, 1.0, 0.1 and 0.1 µg/kg dry weight, respectively. 

#### 4.2.2. Organotin Compounds

The method used to determine the Ots in sediment samples was based on that of Midorikawa *et al.* [30] with some modification. The analytes underwent extraction, centrifugation, elution, cleaning and concentration.

Tributyltin monochloride (TBTCl)-d_27_ and triphenyltin monochloride (TPTCl)-d_15_ were used as a surrogate standard. Tetrabutyltin (TeBT)-d_36_ and tetraphenyltin (TePT)-d_20_ were used as an internal standard. The final solution was concentrated to 0.5 mL. The analytes were detected by GC/MS. The analytical condition is shown in Table 6. The Ot’s detection limits in the sediment samples were set at 0.1 µg/kg dry weight.

**Table 5:** LC/MS/MS conditions (Irgarol 1051, Diuron, Sea-Nine 211, M1).

	Conditions
LC	Agilent model 1100 series HPLC (Agilent; Yokogawa Analytical System, Tokyo, Japan)
Column	narrow bore C18 silica column (2.1 mm i.d. × 150 mm, 5 μm) TSKgel ODS-80T (TOSOH)
Mobile phase	(A):(B) 50:50 → (20 min) → 100:0 (10 min) (A): methanol; (B): water
Flow rate	20 µL/min
Oven temp.	40 °C
Injection vol.	10 µL
MS	PE-Sciex API2000 (Sciex; Applied Biosystems, Framingham, MA, USA)
Analytical mode	ESI-MS-MS
Ionization	positive ion mode
Nitrogen curten gas	40 µL/min
Ion spray voltage	4800 V
Ion source gas 1	40 µL/min
Ion source gas 2	70 µL/min
Collision gas	4 µL/min
Monitor ions	Sea-Nine 211: 282/170(43) Diuron: 233/46(160) Irgarol 1051: 254/198(83) M1: 214/158(43)

**Table 6:** GC/MS conditions (TBT, TPT).

	Conditions
GC	Hewlett-Packard 6890 Series GC System
Column	DB-5ms (0.25mm i.d. × 30m, 0.25 μm) (J&W Scientific Co., Folsom, CA, USA)
Oven temp.	60 °C (2 min)—raised by 20 °C/min—130 °C—raised by 10 °C/min—210 °C—raised by 5 °C/min—260 °C—raised by 10 °C/min—300 °C (2 min)
Flow rate	He, 1 mL/min
	Splitless (purge time 1 min)
Injector temp.	250 °C
Injection vol.	2 µL
MS	(5973N)
Interface temp.	280 °C
Source temp.	230 °C
Ionization energy	280 °C
Source temp.	230 °C
Ionization energy	70 eV
Analytical mode	SIM
Monitor ions	TBT: 263 (261) TPT: 351 (349)

#### 4.2.3. Metals

In a 200-mL beaker, 2 g of wet sediment core sample were mixed with 10 mL of nitric acid and 20 mL of hydrochloric acid while being heated on a hotplate. When the volume had reduced by half, the beaker was removed from the heat. Then, 20 mL of nitric acid was added to the mixture and reheated. When the mixture value had reduced by 20 mL, it was cooled to room temperature. Next, 50 mL of water were added to the mixture, and it was warmed on low heat. After the deposits had settled to the bottom of the beaker, the solution was filtered with filter paper. The resulting solution was used for the analysis of metals, performed on a Thermo electron corporation m series AA Spectrometer with flame. The detection limits of lead, copper, zinc, iron and manganese in the sediment samples were set at 0.5, 0.5, 5.0, 5.0 and 5.0 mg/kg dry weight, respectively.

#### 4.2.4. Chronology

^21^^0^Pb chronology was applied to the cores using the model of Matsumoto [31], in which core depth was corrected for the sediment compaction [32]. Geochronology was also employed based upon the fallout of the fission product, ^137^Cs, from the stratosphere, introduced by nuclear weapons testing. The ^137^Cs profile was adjusted for decay (half-life: 30 y) during the time between the measurement and the sediment deposition, only for the period after 1950, because the ^137^Cs should theoretically not be detected before then. The ^137^Cs maxima (1963 fallout maximum) corroborated the ^21^^0^Pb ages as being reliable.

## 5. Conclusions

1. Irgarol 1051 clearly had the highest concentration measured in four of the five survey points, with survey Point 4 being the exception.

2. Higher concentrations of antifouling biocides in the sediment core samples than the surface sediment samples indicated that this is where accumulation occurs.

3. Oxidation and reduction were not the causes of the high concentration levels of Cu in survey Points 3 and 5, due to the accumulation of Mn or Fe not being detected.

4. The 1990 regulation led to higher concentrations of Ot alternative antifoulants and Ot compounds in survey Point 5. The former was due to increased consumption; the latter due to paint chips containing Ot compounds that were stripped being washed into the sea.

5. After metals have accumulated in the sediment core, they tend not to shift.
